# COVID-19 related mortality in post-operative cardiac surgical patients

**DOI:** 10.1186/s13019-021-01487-x

**Published:** 2021-04-26

**Authors:** Azhar Hussain, Amina Khalil, Priyanka Kolvekar, Prity Gupta, Shyamsunder Kolvekar

**Affiliations:** Department of Cardiac Surgery, St Bartholomew’s Hospital, West Smithfield, London, EC1A 7BE UK

**Keywords:** COVID-19, Cardiac surgery

## Abstract

**Background:**

COVID-19 has caused a global pandemic of unprecedented proportions. Elective cardiac surgery has been universally postponed with only urgent and emergency cardiac operations being performed. The National Health Service in the United Kingdom introduced national measures to conserve intensive care beds and significantly limit elective activity shortly after lockdown.

**Case presentation:**

We report two cases of early post-operative mortality secondary to COVID-19 infection immediately prior to the implementation of these widespread measures.

**Conclusion:**

The role of cardiac surgery in the presence of COVID-19 is still very unpredictable and further studies on both short term and long term outcomes are warranted.

## Introduction

The novel coronavirus, now termed SARS-CoV-2, has caused a significant global impact in the space of 6 months. Elective cardiac surgery has been universally postponed with only urgent and emergency cardiac operations being performed. International guidelines are yet to be defined and almost all healthcare institutions have mandated regional or national measures in place to reduce transmission in susceptible patients. The prevalence of COVID-19 in patients with underlying cardiovascular disease is under-reported, with evidence that pre-existing cardiac disease can render patients more vulnerable to the disease process [1]. The National Health Service in the United Kingdom introduced national measures to conserve intensive care beds and significantly limit elective activity shortly after lockdown [2]. However, these measures were likely implemented after significant viral exposure to the general public and attending healthcare staff. We report two cases of early post-operative mortality secondary to COVID-19 infection immediately prior to the implementation of these widespread measures.

## Case 1

**Fig. 1:**
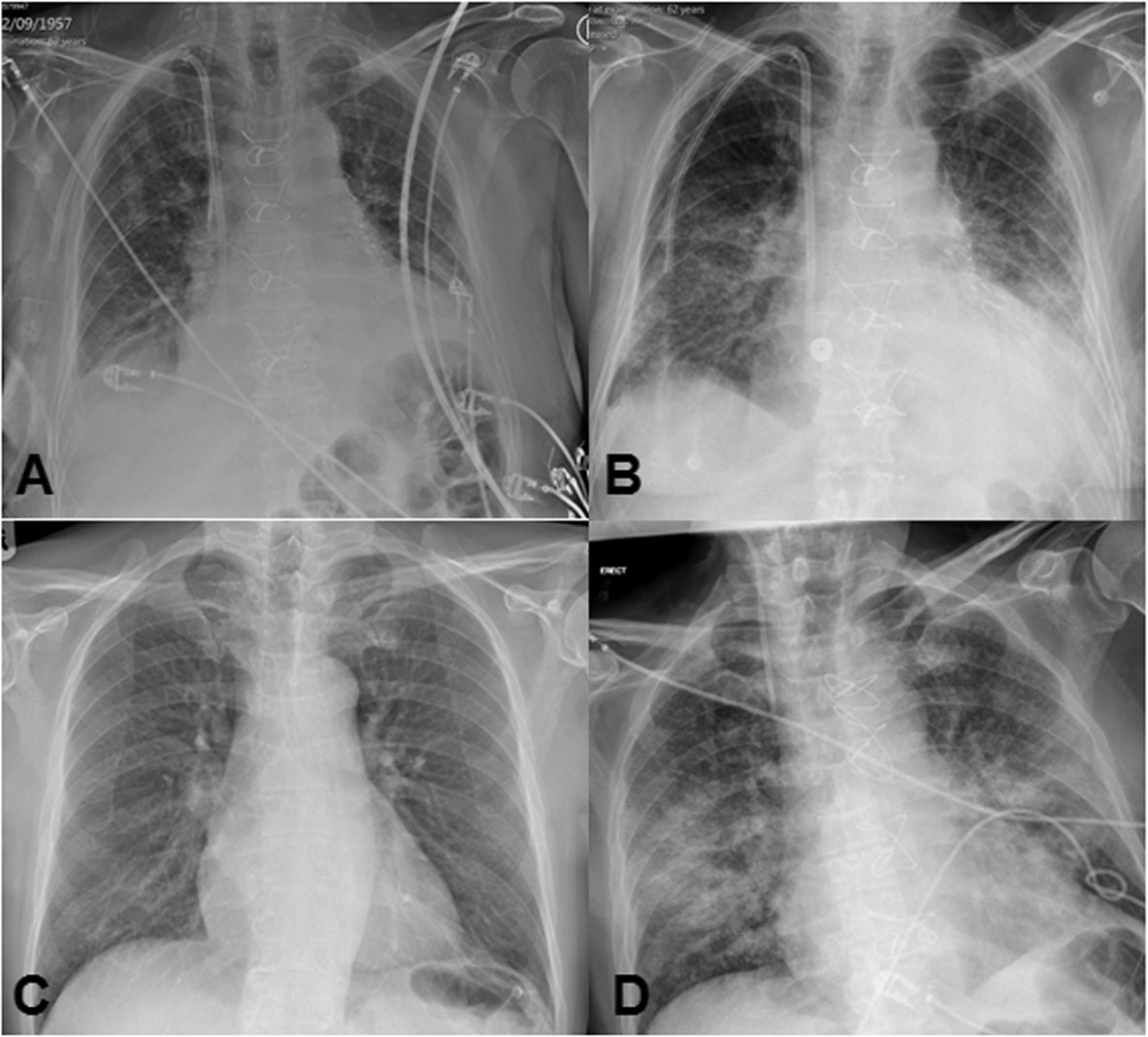
**a** Day 2 post-operative chest XR in case 1 **b** Chest XR of Case 1 on post-operative day 6. **c** Pre-operative XR in case 2. **d** Chest XR of Case 2 on post-operative day 15

A 62-year-old gentleman presented to hospital with increasing chest pain and shortness of breath during renal replacement therapy at his local dialysis centre. His past medical history was significant for end stage renal failure secondary to nephrotic syndrome, hypertension and diabetes mellitus. He was completely independent at home with daily activities prior to admission. Coronary angiography revealed multi vessel disease and a transthoracic echocardiogram showed a preserved left ventricular function with no significant valvular abnormalities. Chest roentgenogram revealed evidence of mild pulmonary oedema. Discussion at the joint cardiology and surgical meeting suggested surgical revascularisation would be the preferred treatment option. He was subsequently transferred to our institution for inpatient coronary artery bypass grafting. A standard median sternotomy was performed with cardiopulmonary bypass established between the ascending aorta and right atrium. The left internal mammary artery and long saphenous vein was harvested to perform three coronary bypass grafts. He was transferred to the intensive care unit post-operatively in a stable condition and stepped down to the ward on the second post-operative day with regular renal specialist input. On the 5th post-operative day the patient reported weakness, rigors and was febrile. A COVID-19 PCR-RNA swab test on the 6th post-operative day was confirmed positive. Chest roentgenogram revealed bilateral lower lobe diffuse opacities, significantly worse from the 2nd post-operative day chest roentgenogram (Fig. 1). He developed acute respiratory failure and was not considered a candidate for mechanical ventilation given his co-morbid conditions and the poor outcomes that were being reported at the time. The patient unfortunately died on the 7th post-operative day with the cause of death being respiratory failure secondary to COVID-19.

## Case 2

A 73-year-old gentleman presented to hospital with progressive shortness of breath and dizziness. His past medical history was significant for polymyalgia rheumatica, hypothyroidism and atrial fibrillation. Initial clinical examination revealed he was febrile with elevated inflammatory markers. He completed a 7-day course of intravenous antibiotics to treat a suspected chest infection with good clinical improvement. Transthoracic echocardiogram revealed a severely dilated left atrium, marked posterior mitral valve prolapse and severe mitral regurgitation. Transesophageal echocardiogram confirmed severe mitral regurgitation with evidence suggestive of infective endocarditis on the posterior leaflet. Coronary angiography revealed proximal left anterior descending artery stenosis. A standard median sternotomy was performed with bicaval and ascending aortic cannulation. Intra-operative examination of the mitral valve revealed large vegetations and perforations on the posterior leaflet. A 31 mm St Jude Medical bioprosthetic valve was implanted in addition to a left internal mammary artery to the left anterior descending artery. The patient was transferred to the intensive care unit in a stable condition and stepped down to the ward on the 2nd post-operative day. He remained an inpatient for the following 9 days post-operatively for intravenous antibiotics as recommended by the endocarditis specialists. He developed a temperature on the 10th post-operative day and a COVID-19 PCR-RNA swab test was confirmed positive. His respiratory requirements increased significantly requiring admission to the intensive care unit for respiratory support. Initial treatment involved non-invasive ventilation however he developed worsening septicaemia with a C-reactive protein of 348 mg/L. Intravenous antibiotics were escalated to meropenem, but unfortunately the patient developed sepsis refractory to inotropic support and multi-organ failure. The patient died on the 18th post-operative day with the cause of death being COVID-19.

## Discussion and conclusion

The prevalence of Sars-CoV-2 in the immediate period prior to national lockdown and widespread changes to medical practice in the healthcare setting is unknown. It is likely, that virus transmission within hospitals was high at a time when adequate personal protective equipment (PPE) was not mandated or simply not feasible due to a lack of supplies. Both patients were deemed ‘routine’ urgent cases and were treated as such in the week prior to implementation of national and regional guidelines to stem viral transmission. The Pan-London Emergency Cardiac Surgery (PLECS) pathway was set up to deliver a regional service for the delivery of urgent and emergency cardiac surgery with a focus on maintaining a COVID-free in-hospital environment [3]. It outlined several measures, including a focus on pre-operative screening and a full theatre operating protocol to protect both staff and patients in our institution.

Our first case highlights some of the rationale behind the many measures that are now currently in place. The decision to operate was initially based on current guidelines which recommend surgical revascularisation in multi-vessel disease in a diabetic patient for best long term outcomes [4]. However, COVID-19 is as yet an unknown disease entity where outcomes in cardiac surgery are unpredictable. Catheter based treatment options were certainly feasible in this case leading to a shorter stay and possibly a better outcome. All patients now referred for surgical treatment currently undergo extensive review to ascertain whether non-surgical options are viable in the first instance, even at the expense of long term outcomes.

Pre-operative COVID-19 testing of patients has now become mandatory in our institution. Our first case did not have a pre-operative COVID-19 swab due to a lack of typical symptoms. Exactly when and where he contracted the virus is difficult to ascertain. He was in regular contact with healthcare professionals due to his dialysis requirements and was an inpatient at our institution at a time where full PPE was not compulsory or deemed required. It is likely that his transmission was iatrogenic given that he developed symptoms at day 7 from his first arrival. The typical incubation period for the virus is between 5 and 10 days [5]. Although this may well have been negative, we also perform CT thorax as part of our pre-operative screening. Recent studies have suggested that CT changes may even precede PCR findings and clinical signs of COVID-19 [6]. This may well have identified early ground-glass changes that were not visible on chest roentgenograms.

Our second case also highlighted the need for aggressive measures to contain intra-hospital spread of the virus. It is almost certain that he contracted the virus whilst at our institution at a time when little to no PPE was used in theatres, in the intensive care unit or on the ward. Although he did warrant urgent inpatient surgery to treat his endocarditis, it is likely that measures put in place now may well have prevented him contracting the virus.

To date, there have been no further mortalities in post-operative cardiac surgical patients secondary to COVID-19 at our institution. This is likely due to the number of measures introduced but also in part due to careful patient selection as well as a significant reduction in surgical volume. The role of cardiac surgery in the presence of COVID-19 is still very unpredictable and further studies on both short term and long term outcomes are warranted.

## Data Availability

Can provide on request.

## Abbreviations

SARS-CoV-2: Severe acute respiratory syndrome coronavirus 2
RNA: Ribonucelic acid
PCR: Polymerase chain reaction
CT: Computed Tomography
PPE: Personal Protective Equiment
